# Assessing Greater Sage-Grouse Selection of Brood-Rearing Habitat Using Remotely-Sensed Imagery: Can Readily Available High-Resolution Imagery Be Used to Identify Brood-Rearing Habitat Across a Broad Landscape?

**DOI:** 10.1371/journal.pone.0156290

**Published:** 2016-05-24

**Authors:** Matthew Westover, Jared Baxter, Rick Baxter, Casey Day, Ryan Jensen, Steve Petersen, Randy Larsen

**Affiliations:** 1 Department of Plant and Wildlife Sciences, Brigham Young University, Provo, Utah, United States of America; 2 Department of Forestry and Natural Resources, Purdue University, West Lafayette, Indiana, United States of America; 3 Geography Department, Brigham Young University, Provo, Utah, United States of America; 4 Monte L. Bean Life Sciences Museum, Brigham Young University, Provo, Utah, United States of America; Oregon State University, UNITED STATES

## Abstract

Greater sage-grouse populations have decreased steadily since European settlement in western North America. Reduced availability of brood-rearing habitat has been identified as a limiting factor for many populations. We used radio-telemetry to acquire locations of sage-grouse broods from 1998 to 2012 in Strawberry Valley, Utah. Using these locations and remotely-sensed NAIP (National Agricultural Imagery Program) imagery, we 1) determined which characteristics of brood-rearing habitat could be used in widely available, high resolution imagery 2) assessed the spatial extent at which sage-grouse selected brood-rearing habitat, and 3) created a predictive habitat model to identify areas of preferred brood-rearing habitat. We used AIC model selection to evaluate support for a list of variables derived from remotely-sensed imagery. We examined the relationship of these explanatory variables at three spatial extents (45, 200, and 795 meter radii). Our top model included 10 variables (percent shrub, percent grass, percent tree, percent paved road, percent riparian, meters of sage/tree edge, meters of riparian/tree edge, distance to tree, distance to transmission lines, and distance to permanent structures). Variables from each spatial extent were represented in our top model with the majority being associated with the larger (795 meter) spatial extent. When applied to our study area, our top model predicted 75% of naïve brood locations suggesting reasonable success using this method and widely available NAIP imagery. We encourage application of our methodology to other sage-grouse populations and species of conservation concern.

## Introduction

Greater sage-grouse (*Centrocercus urophasianus*; hereafter sage-grouse) were determined to be not warranted for protection under the 1973 Endangered Species Act [1]. Populations have decreased steadily since European settlement in western North America [2], and the overall range of sage-grouse has been reduced to 56% of its pre-settlement distribution [3]. The major reasons for the decline include degradation, fragmentation, and loss of sagebrush (*Artemisia* spp.) habitats [4–7]. Sage-grouse are sagebrush obligates and are highly susceptible to changes in sagebrush habitats. Loss or alteration of sagebrush communities has occurred from invasion by native and exotic plants, increased fire frequency and intensity, overgrazing by livestock, energy development, and agricultural or urban development [4, 7–12]. As much as 45% of sagebrush communities that originally existed in western North America have been converted to other landcover types [12]. Consequently, identification of preferred habitat characteristics is necessary to inform conservation and management within remaining sagebrush habitat.

While sage-grouse use diverse sagebrush habitat throughout their life cycle, availability of brood-rearing habitat has been identified as a limiting factor affecting long-term conservation [4, 13–14]. Quality brood-rearing habitat leads to higher chick survival and increased recruitment of chicks into existing populations [15–20]. Previous studies have focused on microsite characteristics [13, 21–29). Studies at these small spatial extents largely shaped contemporary management practices meant to increase quality of brood-rearing habitat. More recently, the focus has shifted to the landscape spatial extent [18, 30–31, 32–34]. With annual home ranges that can be as large as 600 km^2^, examining characteristics of brood-rearing habitat at larger spatial extents is warranted [16, 35–36].

This shift in focus to larger spatial extents has been facilitated by the increased availability and functionality of Geographic Information Systems (GIS) analysis and the widespread availability of multispectral satellite and aerial imagery [37–39]. Recent studies have utilized satellite imagery acquired by the Landsat Thematic Mapper [18] or Enhanced Thematic Mapper [30] satellites. These sensors acquire data at a minimum spatial resolution of 30 m (with the exception of the 15 m resolution panchromatic band which is of limited use for analyses) and a spectral resolution consisting of 7 unique bands across the electromagnetic spectrum [40]. The large spatial resolution of these sensors allows for analysis of expansive areas; however, the minimum unit size for any analyses conducted is also limited by the 30-m spatial resolution. Although Landsat data is collected at relatively short intervals (i.e., weekly or monthly) and has been somewhat useful in understanding habitat selection patterns [24, 30–31], this coarse (≥30m resolution) imagery may not provide the resolution necessary to evaluate habitat characteristics at spatial extents relevant to the process of habitat selection [41].

In addition to the widely and freely available Landsat imagery, there is another dataset available through the National Agricultural Imagery Program (NAIP). This free imagery is collected at approximately 3-year intervals by aerial sensors at a spatial resolution of 1 m and a spectral resolution consisting of 4 unique bands. This fine spatial resolution allows researchers to examine habitat relationships undetectable at the coarser Landsat resolution of 30 m. Using NAIP imagery, factors such as edge effects in highly heterogeneous areas, where patch size is often much smaller than 30 m, can be examined while retaining the capability of assessing large landscapes. In the past, ground-derived microhabitat data were used to assess habitat selection, but its utility was limited [42]. Technology now permits the combination of high-resolution aerial imagery and ground verification, high-resolution images combined together [43], or the combination of high-resolution imagery and ground-based imagery [44] to map habitat and assess habitat selection.

Our goal was to evaluate habitat selection of female sage-grouse with broods across a range of spatial extents utilizing 1 m-resolution NAIP imagery. Our specific objectives were to: 1) determine important features of brood-rearing habitat using fine spatial extent NAIP imagery, 2) assess the spatial extent at which sage-grouse selected of brood-rearing habitat in our study area, and 3) create a predictive habitat model that could be applied across our entire study area to identify areas of preferred brood-rearing habitat. We hypothesized that we would be able to identify features selected by brood-rearing sage-grouse using NAIP imagery and that sage-grouse would select habitat characteristics at multiple spatial extents, as demonstrated in other life history stages for this bird [18].

## Methods

### Study Area

**Fig 1:**
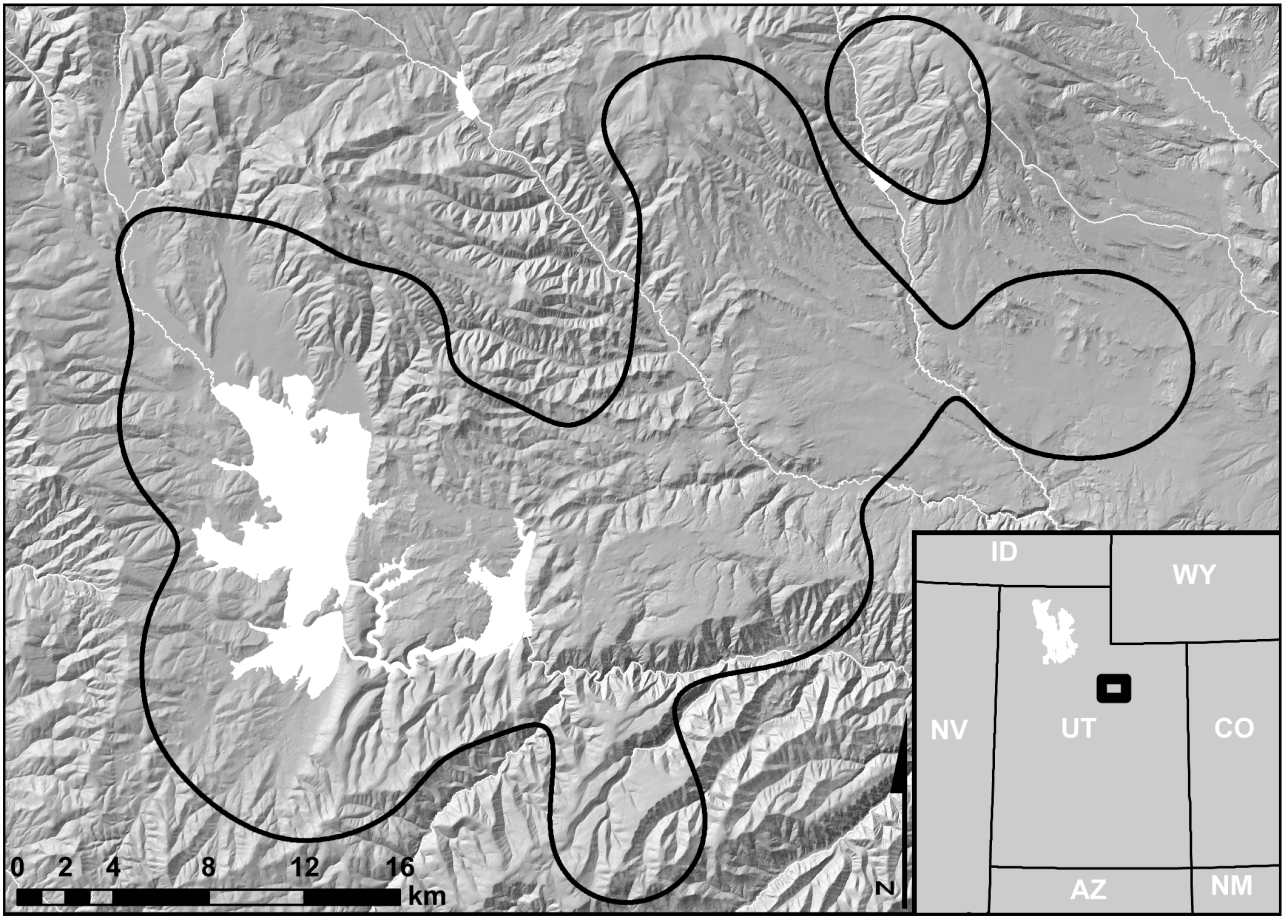
Map of Strawberry Valley in central Utah where we assessed selection of brood-rearing habitat by greater sage-grouse, 1998–2008. The 817 km^2^ study area (polygon outlined in black) was delineated by calculating a 95% confidence polygon of a fixed kernel density estimate using least squares cross validation (LSCVh) to select the smoothing parameter (h) on 3,865 locations of female sage-grouse collected from 1998 to 2008. White areas represent water.

Our study area was an 817 km^2^ area surrounding Strawberry Reservoir in north-central Utah (Fig 1). We delineated this area by running a fixed-kernel density estimator (using least-squares cross validation (LSCVh) to select the smoothing parameter (h)) on 3,865 locations (nest, brood, and non-brood) of female sage-grouse collected from 1998 to 2008. We then used Home Range Tools (http://www.blueskytelemetry.com) for ArcGIS version 9.3^®^ (ESRI, Inc., Redlands, CA) to create a 95% polygon surrounding these locations. This polygon (Fig 1) contained 824 of 836 (98.5%) brood locations in our dataset. We removed the remaining 12 brood locations from our analysis, considering them to be outliers.

The study area defined by the LSCVh fixed-kernel density estimate was a high mountain valley that transitioned to lower elevations moving eastward. Elevation ranged from 1,946 to 3,150 m. Average annual precipitation varied widely from 43 cm in the lower elevations to 84 cm at the highest elevations (www.ncdc.noaa.gov). Vegetation consisted of shrublands dominated by big sagebrush (*A*. *tridentata*). Silver sagebrush (*A*. *cana*) occurred in the more mesic areas and black greasewood (*Sarcobatus vermiculatus*) was found in some areas in the eastern part of the study area at lower elevations. On slopes at higher elevations, tree communities consisted of quaking aspen (*Populus tremuloides*), Gambel’s oak (*Quercus gambelii*), and various conifers (e.g. *Abies* spp., *Picea* spp., and *Pseudotsuga* spp.). The tree community at lower elevations was dominated by juniper (*Juniperus* spp.) with scattered pinyon pine (*Pinus edulis*). Common forbs found in the study area included longspur lupine (*Lupinus arbustus*), silky lupine (*Lupinus sericeus*), sticky purple geranium (*Geranium viscosissimum*), and sulphur-flower buckwheat (*Eriogonum umbellatum*). Common grasses included Kentucky bluegrass (*Poa pratensis*), and smooth brome (*Bromus inermis*). In the lower elevations, cheatgrass (*Bromus tectorum*) occurred in the understory, but this exotic species was largely absent from the study area. Riparian areas were dominated by willow species (*Salix* spp). No fires occurred within our study area during our study years and grazing by domestic livestock was absent. Similarly, habitat enhancement via mechanical removal of sagebrush or pinyon-juniper did not occur in the study area until 2009.

### Data Collection

We captured female sage-grouse annually by netting them with the aid of all-terrain vehicles on and around leks during the months of March through May using a modified spotlighting method [45]. Once captured, we fitted sage-grouse with necklace style radio transmitters (Advanced Telemetry Systems, Inc., Isanti, MN) and tracked them using a 4-element Yagi antenna and either a Telonics TR 2 (Telonics, Inc., Mesa, AZ) or Communication Specialists R-1000 (Communication Specialists, Inc., Orange, CA) digital telemetry receiver. We monitored females approximately twice weekly from April through August. Nest initiation occurred in late April or May for females in our study area. Females that hatched at least one egg and were observed with at least one chick, were considered brooding. We monitored broods at least twice weekly during daytime hours through 56 days post-hatch. We also opportunistically encountered brooding and non-brooding females during the study. If we were unable to visually detect chicks with a female, we left the immediate area and observed the location for 20 minutes or until the female returned. We classified females that flew long distances and did not return to the area, or were located twice consecutively without chicks as non-brooding and did not include them in our brood sample. For more information on trapping and collection of telemetry data see Baxter et al. 2008 [46] or Peck 2011 [47]. Our sample of broods also included those associated with sage-grouse translocated from four neighboring populations. Because these sage-grouse flocked with resident grouse and demonstrated little to no difference in movements, reproduction, or survival, we lumped their locations with those of resident grouse [46, 48].

### Ethics Statement

Trapping and handling of sage-grouse was permitted and approved by the Utah Division of Wildlife Resources under a Certificate of Registration (#1COLL6817) and by Brigham Young University’s Institutional Animal Care and Use Committee (IACUC approval #08–0402).

### Imagery Classification

To characterize vegetation in our study area, we performed a supervised classification on 1-m resolution NAIP imagery collected in 2006. This year represented the first that statewide coverage of 1-m resolution imagery was available for Utah. Thereafter, NAIP imagery was collected every 3 years. Although we could have benefited from imagery collected more frequently—particularly during the early years of our study—Strawberry Valley experienced very little of the habitat change that has impacted sagebrush systems in much of western North America. Consequently, we viewed any potential bias associated with collection of sage-grouse locations in years before or after the 2006 image as unlikely to influence our results. We used ENVI EX Feature Extraction^®^ (Exelis Visual Information Solutions, Inc. McLean, VA) to classify NAIP imagery. Using this classification, as well as digitization to manually identify road classes in ArcGIS version 10^®^ (ESRI, Inc., Redlands, CA), we generated a landcover layer that divided the landscape into the following 10 classes: paved roads, high-use or major dirt roads (graveled/wide enough for two-way traffic), low-use or minor dirt roads (two tracks), bare soil, shrubs, trees, grass, water, riparian areas, and agricultural areas. Our shrub landcover class consisted of almost entirely sagebrush species; however, due to the limited spectral bands available in NAIP imagery we were unable to differentiate between species. In order to ensure the accuracy of our landcover layer and prior to assessment of sage-grouse selection, we performed an on-the-ground accuracy assessment. Using ArcGIS 10, we randomly distributed 502 points across the study area. In the summer of 2011, we visited 202 of these points and recorded which of the 10 landcover classes best described each location. Using this information and our aerial imagery, we visually interpolated the landcover classes for the remaining 300 locations that we were unable to access for a variety of reasons (e.g. private property). We then used these data (S1 File) to calculate accuracy statistics for our landcover classification [49].

### Statistical Analysis

Following accuracy assessment, we developed a list of 86 explanatory variables (Table 1) that may have influenced selection of brood-rearing habitat by sage-grouse in our area based on previous literature [13, 18, 21–31] and our own experience in the study area since 1998. We then divided the variables into two groups: those that would be best examined at multiple spatial extents, and those for which a single spatial extent was adequate. Variables that we evaluated at a single spatial extent (n = 44) included distances to various features and variables for which only the values at the actual use or random site were relevant (Table 1). The remaining 42 variables (Table 2) were dependent on spatial extent. For variables dependent on spatial extent, we calculated values at three spatial extents by generating circular buffers with radii of 45, 200, and 795 m surrounding each site. The 200 and 795-m spatial extents were selected based on the lower and upper end of daily brood movements [22]. The 45-m spatial extent was representative of common patch sizes available to broods in our study area. Prior to modeling, we tested for multicollinearity between explanatory variables and did not combine in a single model any variables with a correlation coefficient > |0.6|.

**Table 1:** Data used to model brooding habitat selection by female sage-grouse from 1998 to 2008 in Strawberry Valley, Utah, USA.

Variable Name	Description
DistAgriculture	Distance to the landcover class "agriculture"
Aspect	Aspect of the cell containing the point (10m resolution)
JuneSolar	Total solar radiation received by the cell containing the point during the month of June (10m resolution)
JulySolar	Total solar radiation received by the cell containing the point during the month of July (10m resolution)
AugustSolar	Total solar radiation received by the cell containing the point during the month of August (10m resolution)
DistCamp	Distance to common campsites (both improved and non-improved)
SlopeDegrees	Slope of the cell containing the point in degrees (10m resolution)
DistEdgeGrassRiparar	Distance to edge consisting of grass on one side and riparian on the other
DistEdgeGrassShrub	Distance to edge consisting of grass on one side and shrub on the other
DistEdgeGrassSoil	Distance to edge consisting of grass on one side and bare soil on the other
DistEdgeGrassTree	Distance to edge consisting of grass on one side and tree on the other
DistEdgeGrassWater	Distance to edge consisting of grass on one side and water on the other
DistMinDirtRoad	Distance to the landcover class "minor dirt road"
DistHabEdge	Distance to any type of habitat edge
DistRiparShrub	Distance to edge consisting of riparian on one side and shrub on the other
DistRiparSoil	Distance to edge consisting of riparian on one side and bare soil on the other
DistRiparTree	Distance to edge consisting of riparian on one side and tree on the other
DistRiparWater	Distance to edge consisting of riparian on one side and water on the other
DistEdgeShrubSoil	Distance to edge consisting of shrub on one side and bare soil on the other
DistEdgeShrubTree	Distance to edge consisting of shrub on one side and tree on the other
DistEdgeShrubWater	Distance to edge consisting of shrub on one side and water on the other
DistEdgeSoilTree	Distance to edge consisting of bare soil on one side and tree on the other
DistEdgeTreeWater	Distance to edge consisting of tree on one side and water on the other
DistEdgeWaterSoil	Distance to edge consisting of water on one side and bare soil on the other
Elevation	Elevation of the cell containing the point (10m resolution)
DistGrass	Distance to the landcover class "grass"
DistMajDirtRoad	Distance to the landcover class "major dirt road"
DistLake	Distance to the landcover class "standing water"
PatchSize	Size of the patch containing the point
DistPavedRoad	Distance to the landcover class "paved road"
DistPermStruct	Distance to any permanent structure
DistTransmissionLine	Distance to above-ground transmission lines
DistRipar	Distance to the landcover class "riparian"
DistStream	Distance to the landcover class "flowing water"
DistShrub	Distance to the landcover class "shrub"
DistSoil	Distance to the landcover class "bare soil"
TopoIndex100	Topographic Position Index calculated with a circular neighborhood of 100 cells (Jenness 2011)
TopoIndex200	Topographic Position Index calculated with a circular neighborhood of 200 cells (Jenness 2011)
TopoIndex300	Topographic Position Index calculated with a circular neighborhood of 300 cells (Jenness 2011)
DistTree	Distance to the landcover class "tree"
Ruggedness159	Vector Ruggedness Measure calculated with a square neighborhood of 159 cells (Sappington et al 2007)
Ruggedness25	Vector Ruggedness Measure calculated with a square neighborhood of 25 cells (Sappington et al 2007)
Ruggedness9	Vector Ruggedness Measure calculated with a square neighborhood of 9 cells (Sappington et al 2007)
Ruggedness3	Vector Ruggedness Measure calculated with a square neighborhood of 3 cells (Sappington et al 2007)

**Table 2:** List of 42 spatial extent variant variables used to build models of brood-habitat selection for greater sage-grouse in Strawberry Valley, UT, 1998–2008. For these variables, we calculated values for each at three different spatial extents by generating a circle with radii of 45, 200, and 795 m surrounding each use or random site and then summarized attributes classified from 1 m resolution (NAIP) aerial imagery.

Variable Name	Description
TreeCover	The proportion of the "tree" landcover class in a circular buffer
SoilCover	The proportion of the "bare soil" landcover class in a circular buffer
ShrubCover	The proportion of the "shrub" landcover class in a circular buffer
GrassCover	The proportion of the "grass" landcover class in a circular buffer
RiparCover	The proportion of the "riparian" landcover class in a circular buffer
MajDirtRoadCover	The proportion of the "major dirt road" landcover class in a circular buffer
MinDirtRoadCover	The proportion of the "minor dirt road" landcover class in a circular buffer
PavedRoadCover	The proportion of the "paved road" landcover class in a circular buffer
AgricultureCover	The proportion of the "agriculture" landcover class in a circular buffer
LakeCover	The proportion of the "standing water" landcover class in a circular buffer
StreamCover	The proportion of the "flowing water" landcover class in a circular buffer
EdgeAgricultureGrass	Meters of edge consisting of agriculture on one side and grass on the other in a circular buffer
EdgeAgricultureRipar	Meters of edge consisting of agriculture on one side and riparian on the other in a circular buffer
EdgeAgricultureShrub	Meters of edge consisting of agriculture on one side and shrub on the other in a circular buffer
EdgeAgricultureSoil	Meters of edge consisting of agriculture on one side and bare soil on the other in a circular buffer
EdgeAgricultureTree	Meters of edge consisting of agriculture on one side and tree on the other in a circular buffer
EdgeGrassRipar	Meters of edge consisting of grass on one side and riparian on the other in a circular buffer
EdgeGrassShrub	Meters of edge consisting of grass on one side and shrub on the other in a circular buffer
EdgeGrassSoil	Meters of edge consisting of grass on one side and soil on the other in a circular buffer
EdgeGrassTree	Meters of edge consisting of grass on one side and tree on the other in a circular buffer
EdgeGrassWater	Meters of edge consisting of grass on one side and water on the other in a circular buffer
EdgeMajDirtRoadGrass	Meters of edge consisting of major dirt road on one side and grass on the other in a circular buffer
EdgeMajDirtRoadRipar	Meters of edge consisting of major dirt road on one side and riparian on the other in a circular buffer
EdgeMajDirtRoadShrub	Meters of edge consisting of major dirt road on one side and shrub on the other in a circular buffer
EdgeMajDirtRoadTree	Meters of edge consisting of major dirt road on one side and tree on the other in a circular buffer
EdgeMinDirtRoadGrass	Meters of edge consisting of minor dirt road on one side and grass on the other in a circular buffer
EdgeMinDirtRoadRipar	Meters of edge consisting of minor dirt road on one side and riparian on the other in a circular buffer
EdgeMinDirtRoadShrub	Meters of edge consisting of minor dirt road on one side and shrub on the other in a circular buffer
EdgeMinDirtRoadTree	Meters of edge consisting of minor dirt road on one side and tree on the other in a circular buffer
EdgePavedRoadRipar	Meters of edge consisting of paved road on one side and riparian on the other in a circular buffer
EdgePavedRoadShrub	Meters of edge consisting of paved road on one side and shrub on the other in a circular buffer
EdgePavedRoadSoil	Meters of edge consisting of paved road on one side and bare soil on the other in a circular buffer
EdgePavedRoadTree	Meters of edge consisting of paved road on one side and tree on the other in a circular buffer
EdgeRiparShrub	Meters of edge consisting of riparian on one side and shrub on the other in a circular buffer
EdgeRiparSoil	Meters of edge consisting of riparian on one side and bare soil on the other in a circular buffer
EdgeRiparTree	Meters of edge consisting of riparian on one side and tree on the other in a circular buffer
EdgeShrubSoil	Meters of edge consisting of shrub on one side and bare soil on the other in a circular buffer
EdgeShrubTree	Meters of edge consisting of shrub on one side and tree on the other in a circular buffer
EdgeShrubWater	Meters of edge consisting of shrub on one side and water on the other in a circular buffer
EdgeSoilTree	Meters of edge consisting of bare soil on one side and tree on the other in a circular buffer
EdgeTreeWater	Meters of edge consisting of tree on one side and water on the other in a circular buffer
EdgeWaterSoil	Meters of edge consisting of water on one side and bare soil on the other in a circular buffer

To determine the variables that best differentiated use from random sites, we used a multi-staged information theoretic approach [50] within a mixed-effects logistic regression [51], using a random intercept to account for individual heterogeneity. We scaled all variables to have a mean of zero and a standard deviation of one prior to analysis. We then used ArcGIS 10 to calculate values for all of our explanatory variables at each spatial extent for 675 brood locations collected from radio-marked females between 1998 and 2008 (remaining locations collected between 1998 and 2008, n = 149, were from unmarked females and we withheld them for accuracy assessment along with locations collected between 2009 and 2012). We then generated an equal number of random (i.e. available) locations from within the study area after masking out Strawberry Reservoir. Because our random locations were cast within the boundary of the study area and not associated with individual home ranges, our modeling of resource selection generally corresponded to Johnson’s 2^nd^ order of selection [52] To ensure that 675 random locations adequately characterized our study area, we calculated the true mean (i.e. mean of all pixels/resource units) for continuous variables and compared our sample means with 95% CIs to these values [53]. In every case, the confidence intervals and even standard errors of our sample overlapped the true mean values suggesting that 675 random locations was adequate to characterize our study area.

Next, we developed 35 *a priori*, univariable and multivariable models (Table 3) and used model selection within each of our three spatial extents based on previous literature [13, 18, 21–31] and our own experience (>15 years) to determine which variables best differentiated use from random locations [51]. To evaluate relative model support, we judged models based on minimization of Akaike’s Information Criterion (AIC) [54]. We followed this same procedure for the spatial extent invariant variables with a set of 34 a priori models (Table 4). For each of these four groups (45, 200, 795-m spatial extents and spatial extent invariant variables), we advanced the top model and any competitive models (≤ 2 ΔAIC) to a second stage of analysis.

**Table 3:** List of 35 brood-habitat selection models developed for analysis of spatial extent variant variables for greater sage-grouse in Strawberry Valley, UT, 1998–2008. Descriptions of variables are found in Table 2.

Model	Structure
1	Use ~ EdgeAgricultureGrass, EdgeAgricultureRipar, EdgeAgricultureShrub, EdgeAgricultureSoil, EdgeAgricultureTree
2	Use ~ EdgeAgricultureGrass, EdgeGrassRipar, EdgeGrassShrub, EdgeGrassSoil, EdgeGrassTree, EdgeGrassWater, EdgeMajDirtRoadGrass, EdgeMinDirtRoadGrass
3	Use ~ EdgeShrubTree, EdgeRiparTree
4	Use ~ TreeCover
5	Use ~ SoilCover
6	Use ~ ShrubCover
7	Use ~ GrassCover
8	Use ~ RiparCover
9	Use ~ MajDirtRoadCover
10	Use ~ MinDirtRoadCover
11	Use ~ PavedRoadCover
12	Use ~ AgricultureCover
13	Use ~ LakeCover
14	Use ~ StreamCover
15	Use ~ RiparCover, LakeCover, StreamCover
16	Use ~ ShrubCover, GrassCover, RiparCover
17	Use ~ RiparCover, ShrubCover
18	Use ~ MajDirtRoadCover, MinDirtRoadCover, PavedRoadCover
19	Use ~ MajDirtRoadCover, MinDirtRoadCover, PavedRoadCover, AgricultureCover
20	Use ~ TreeCover, SoilCover
21	Use ~ EdgeShrubSoil, EdgeAgricultureShrub, EdgeShrubWater, EdgeShrubTree, MajDirtSag, MinDirtSag, EdgePavedRoadShrub
22	Use ~ EdgeMajDirtRoadGrass, EdgeMajDirtRoadRipar, MajDirtSag, EdgeMajDirtRoadTree
23	Use ~ EdgeMinDirtRoadGrass, EdgeMinDirtRoadRipar, MinDirtSag, EdgeMinDirtRoadTree
24	Use ~ EdgePavedRoadRipar, EdgePavedRoadShrub, EdgePavedRoadSoil, EdgePavedRoadTree
25	Use ~ EdgeRiparShrub, EdgeRiparSoil, EdgeRiparTree, EdgePavedRoadRipar, EdgeAgricultureRipar, EdgeMajDirtRoadRipar, EdgeMinDirtRoadRipar
26	Use ~ EdgeWaterSoil, EdgeRiparSoil, EdgeSoilTree, EdgeShrubSoil, EdgePavedRoadSoil
27	Use ~ EdgeRiparShrub, EdgeGrassRipar, EdgeShrubWater
28	Use ~ EdgeShrubTree, EdgeRiparTree, EdgeGrassTree, EdgePavedRoadShrub, MajDirtSag
29	Use ~ ShrubCover, GrassCover, RiparCover, TreeCover
30	Use ~ ShrubCover, GrassCover, RiparCover, TreeCover, PavedRoadCover
31	Use ~ ShrubCover, GrassCover, RiparCover, TreeCover, PavedRoadCover, MajDirtRoadCover, MinDirtRoadCover
32	Use ~ ShrubCover, GrassCover, RiparCover, TreeCover, PavedRoadCover, MajDirtRoadCover
33	Use ~ ShrubCover, GrassCover, RiparCover, PavedRoadCover, EdgeShrubTree, EdgeRiparTree
34	Use ~ ShrubCover, GrassCover, RiparCover, TreeCover, PavedRoadCover, EdgeShrubTree
35	Use ~ ShrubCover, GrassCover, RiparCover, PavedRoadCover, EdgeShrubTree

**Table 4:** List of 35 brood-habitat selection models developed for analysis of spatial extent invariant variables for greater sage-grouse in Strawberry Valley, Utah, 1998–2008. Descriptions of variables are found in Table 1.

Model	Structure
1	Use ~ DistAgriculture
2	Use ~ Aspect + SlopeDegrees + Elevation
3	Use ~ JuneSolar
4	Use ~ JulySolar
5	Use ~ AugustSolar
6	Use ~ DistCamp + DistPermStruct
7	Use ~ DistCamp + DistPermStruct + DistAgriculture
8	Use ~ DistEdgeGrassRiparar
9	Use ~ DistEdgeGrassShrub
10	Use ~ DistRiparShrub
11	Use ~ DistHabEdge
12	Use ~ DistEdgeShrubTree + DistRiparTree
13	Use ~ DistGrass + DistRipar + DistShrub
14	Use ~ DistRipar + DistShrub
15	
16	Use ~ DistMajDirtRoad + DistPavedRoad
17	Use ~ DistCamp + DistPermStruct + DistAgriculture + DistPavedRoad
18	Use ~ DistMajDirtRoad + DistPavedRoad + DistMinDirtRoad
19	Use ~ DistRipar + DistLake + DistStream
20	Use ~ DistLake + DistStream
21	Use ~ DistShrub
22	Use ~ DistRipar
23	Use ~ DistTree
24	Use ~ DistTree + DistTransmissionLine
25	Use ~ PatchSize
26	Use ~ TopoIndex100
27	Use ~ TopoIndex200
28	Use ~ TopoIndex300
29	Use ~ Ruggedness159
30	Use ~ Ruggedness25
31	Use ~ Ruggedness3
32	Use ~ Ruggedness9
33	Use ~ DistEdgeGrassRiparar + DistEdgeGrassShrub + DistRiparShrub
34	Use ~ DistTransmissionLine
	Use ~ DistTree + DistTransmissionLine + DistPermStruct

In our second stage of analysis, we combined the models that were advanced from stage 1 into 7 new models (Table 5). We created these models by combining the top models from each spatial extent with the variables in the top model from the spatial extent invariant group. In these 7 models, for spatial extent dependent variables, we used the spatial extent at which the univariable model for that variable had the lowest AIC in the first stage of analysis.

**Table 5:** Model rankings (AIC and ΔAIC), model weights (*w*_*i*_), number of estimated parameters (*K*), and log likelihood (LL) for supported (model weight ≥ 1%) models of greater sage-grouse selection of brood-rearing habitat in Strawberry Valley, UT, 1998–2008 at three spatial extents and for spatial extent-invariant variables.

Model^a^	Structure	AIC	ΔAIC	*w*_*i*_	*K*	LL
45 meters						
33	Use~ShrubCover + GrassCover + RiparCover + PavedRoadCover + EdgeShrubTree + RiparianTree	1456.2	0	0.86	8	-720.07
35	Use~ShrubCover + GrassCover + RiparCover + PavedRoadCover + EdgeShrubTree	1460.5	4.2	0.10	7	-723.18
200 meters						
33	Use~ShrubCover + GrassCover + RiparCover + PavedRoadCover + EdgeShrubTree + RiparianTree	1298.8	0	1.00	8	-641.36
795 meters						
34	Use~ShrubCover + GrassCover + RiparCover + TreeCover + PavedRoadCover + EdgeShrubTree	1178.1	0	0.77	8	-581.01
35	Use~ShrubCover + GrassCover + RiparCover + PavedRoadCover + EdgeShrubTree	1181.5	3.4	0.14	7	-583.73
33	Use~ShrubCover + GrassCover + RiparCover + PavedRoadCover + EdgeShrubTree + EdgeRiparTree	1182.4	4.3	0.09	8	-583.16
Spatial extent Invariant						
35	Use~DistTree + DistTransmissionLine + DistPermStruct	1537.6	0	0.74	5	-763.78
24	Use~DistTree + DistTransmissionLine	1539.8	2.2	0.25	4	-765.87

^a^ Model numbers correspond to those in Tables 3 and 4.

We evaluated beta coefficients based on their standard errors and 85% confidence intervals [52]. To evaluate effect sizes for variables in our top models, we calculated a resource selection function (RSF) by holding all other variables constant at their mean. We used variance inflation factors (VIF) to test for multicollinearity among variables in our final models. We considered VIF > 5 to indicate multicollinearity. To assess predictive ability of our final models, we performed a k-folds cross validation [52] where k = 5. We sorted the data into 5 partitions, with an approximately equal number of locations in each partition. In each iteration of our procedure, four partitions (80% of the data) were used as the training set, while the remaining partition (20% of the data) was used as the test set. We repeated this procedure until all data were used both as the test set and as part of the training set. We regressed the number of locations from the test (used) dataset in each bin against the median RSF value of the random locations in each bin. We report mean coefficient of determination and slope, and we considered the combination of a high coefficient of determination and a positive slope to be indicative of a model that differentiated between use and available locations well [55].

## Results

Our NAIP classification showed composition of landcover in our study to be 45.5% shrubs, 28.7% trees, 11.9% grass, 7.6% water, 3.0% bare soil, 1.7% riparian, 0.7% agriculture, 0.7% low-use dirt roads, 0.1% paved roads, and 0.1% high-use dirt roads. Accuracy assessment of our classification [49] yielded an overall accuracy of 78.5% and a Kappa value of 0.707. We utilized 675 relocations of sage-grouse broods from 120 females over 15 years. The number of locations per female ranged between 1–28, with a mean of 5.6 locations per female. Unique females were relocated from 1–3 years depending on battery life of transmitters and length of time each bird survived.

The top-ranked model at the 45-m spatial extent included the combination of percent shrub, percent grass, percent riparian, percent paved road, meters of shrub/tree edge, and meters of riparian/tree edge (Table 5). There were no other competitive models. Results from the 200-m spatial extent were the same as the 45-m spatial extent (Table 5). At the 795-m spatial extent, we had three competitive models. The top model included the combination of percent shrub, percent grass, percent riparian, percent paved road, percent tree, and meters of shrub/tree edge. A similar model without percent tree had a ΔAIC of 3.4 (Table 5) and only accounted for 14% of model weight. The spatial extent-invariant group also had two competitive models. The top model consisted of distance to trees, distance to transmission lines, and distance to permanent structures. The next best competitive model, with a ΔAIC of 2.2 and 25% of model weight, was the same as the top model with the exclusion of distance to permanent structures (Table 5).

**Fig 2:**
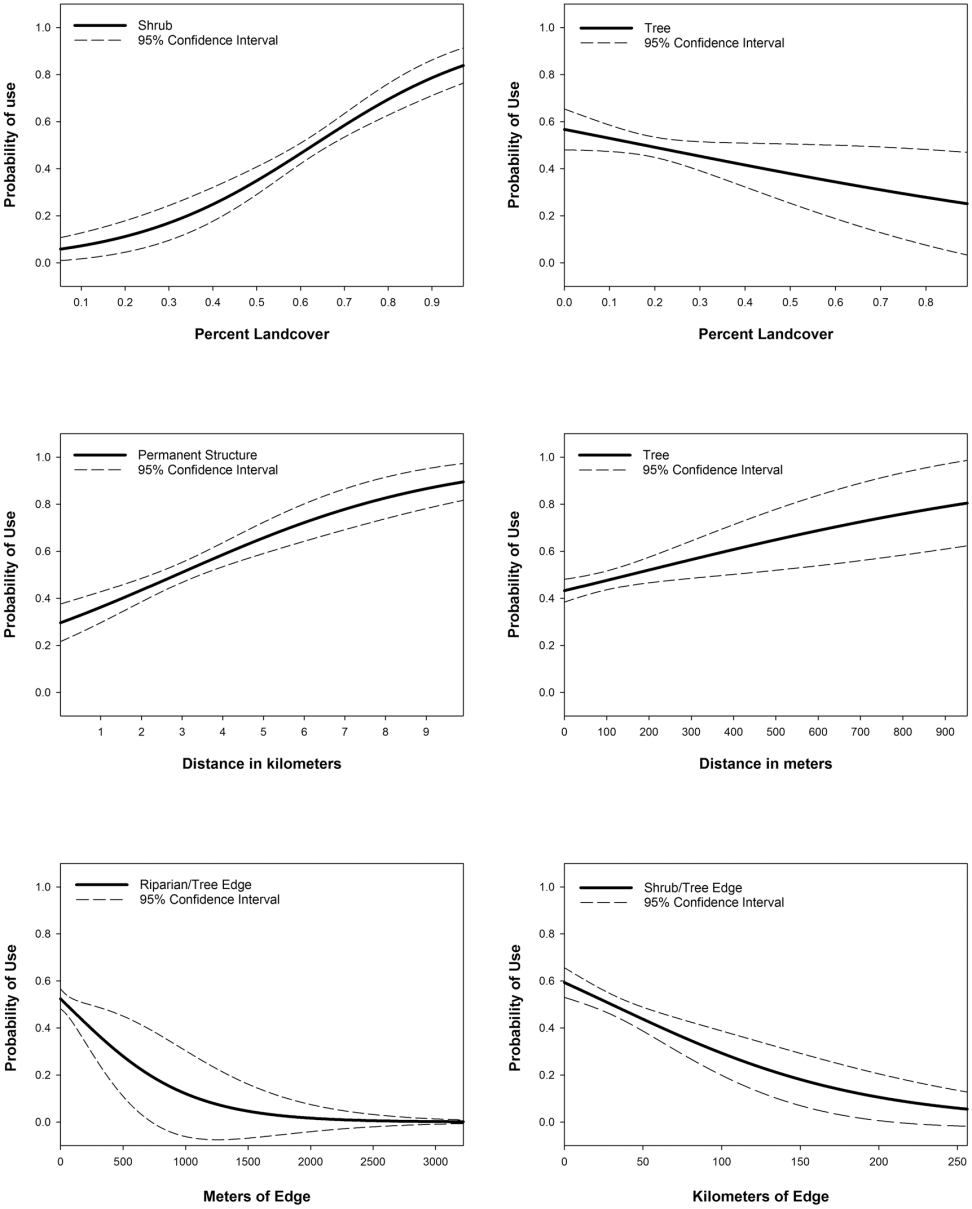
Resource selection functions for selected variables in the top model showing relative probability of use in relation to explanatory variables for greater sage-grouse broods in Strawberry Valley, UT, 1998–2008.

In the second stage of analysis our top model consisted of 5 variables from the 795-m spatial extent (percent shrub, percent grass, percent tree, percent paved road, and meters of sage/tree edge), 1 from the 200-m spatial extent (meters of riparian/tree edge), 1 from the 45-m spatial extent (percent riparian), and 3 from the spatial extent invariant group (distance to tree, distance to transmission lines, and distance to permanent structures) (Table 6). This model suggested that 7 variables were negatively correlated with selection of brood-rearing habitat based on their coefficients and RSFs: percent grass, percent tree, percent paved road, meters of shrub/tree edge, meters of riparian/tree edge, and increased distance from transmission lines (Fig 2; Table 7). The remaining 3 variables were positively correlated with selection: percent shrub, percent riparian, distance to tree, and distance to permanent structure (Fig 2).

**Table 6:** Akaike’s information criterion (AIC and ΔAIC) selected models, model weights (*w*_*i*_), number of estimated parameters (*K*), and log likelihood (LL) for supported (model weight ≥ 1%) models from the combined analysis of sage-grouse selection for brood-rearing habitat in Strawberry Valley, UT, 1998–2008.

Model^a^	Structure	AIC	ΔAIC	*w*_*i*_	*K*	LL
6	795ShrubCover + 795GrassCover + 45RiparCover + 795TreeCover + 795PavedRoadCover + 795EdgeShrubTree + 200EdgeRiparTree + DistTree + DistTransmissionLine	1145.0	0	0.80	11	-561.42
3	795ShrubCover + 795GrassCover + 795RiparCover + 795TreeCover + 795PavedRoadCover + 795EdgeShrubTree + DistTree + DistTransmissionLine	1147.9	2.8	0.19	10	-563.85
Null	Constant	1875.5	730.5	0.00	2	-935.75

^a^Model numbers correspond to those in Tables 3 and 4.

**Table 7:** Estimated coefficients (β-estimate), standard errors (SE), and 85% confidence intervals for variables in our final model-averaged model.

				85% CI	
Variable description	Variable	β-estimate	SE	Lower	Upper
Percent Shrub, 795-m extent	X795ShrubCover	1.09	0.19	0.82	1.36
Percent Grass, 795-m extent	X795GrassCover	-0.71	0.11	-0.87	-0.55
Percent Riparian, 45-m extent	X45RiparCover	0.07	0.10	-0.07	0.21
Percent Tree, 795-m extent	X795TreeCover	-0.30	0.20	-0.58	-0.01
Percent Paved Road, 795-m extent	X795PavedRoadCover	-0.42	0.08	-0.54	-0.31
Meters of sage-tree edge, 795-m extent	X795SageTree	-0.46	0.12	-0.63	-0.29
Meters of riparian-tree edge, 200-m extent	X200EdgeRiparTree	-0.46	0.19	-0.73	-0.18
Meters to trees	DistTree	0.47	0.14	0.27	0.67
Meters to power lines	DistTransmissionLine	-0.32	0.08	-0.44	-0.20
Percent Riparian, 795-m extent	X795RiparCover	-0.18	0.10	-0.32	-0.05

**Fig 3:**
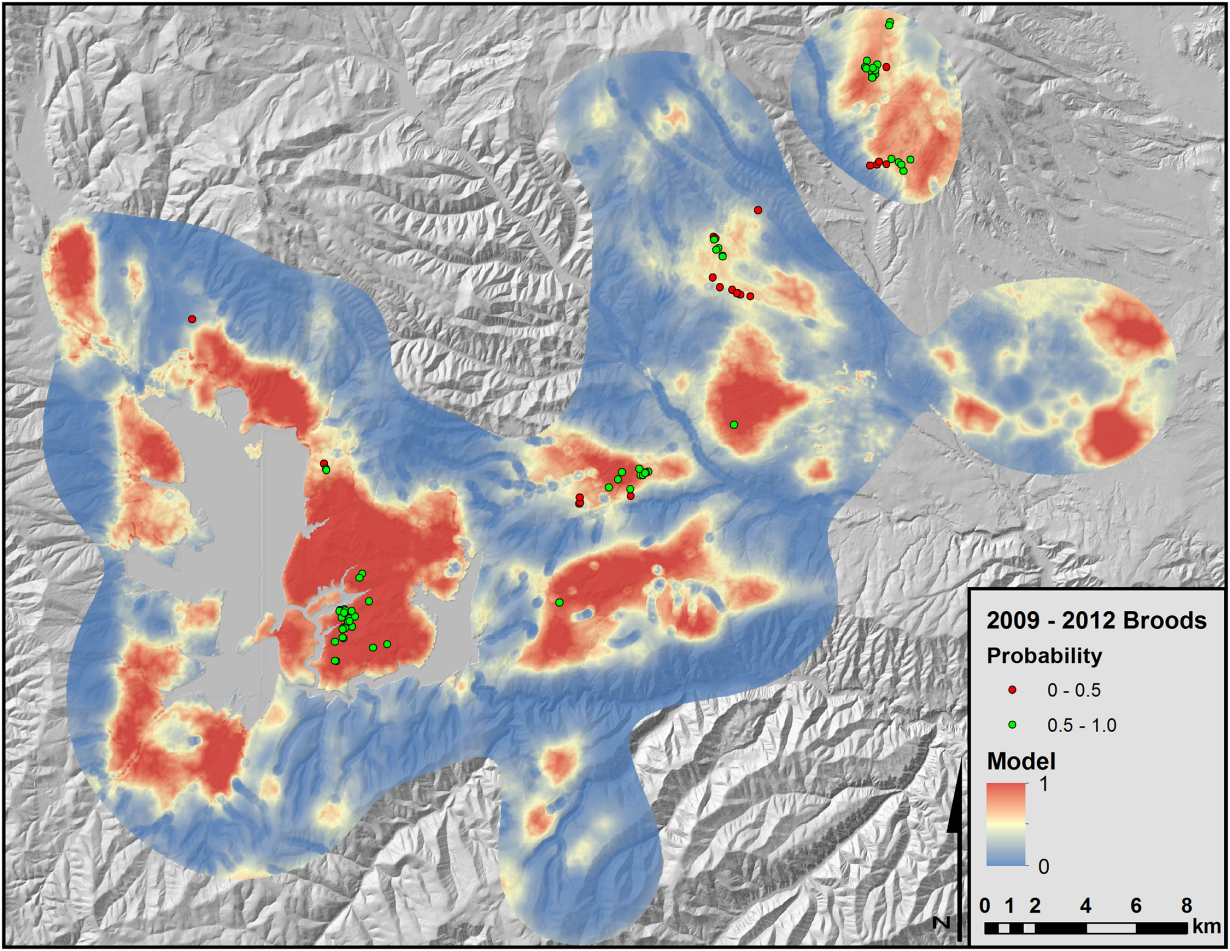
Predicted brood-rearing habitat for greater sage-grouse in Strawberry Valley, Utah based on logistic regression models of landscape features identified using 1-m NAIP imagery. Data used to generate the predictive model were collected between 1998 and 2008. Locations used to verify accuracy of the habitat map were collected between 2009 and 2012. Grey areas represent water.

From the k-folds cross validation, our mean adjusted r-squared was 0.96, mean slope was 17.67, and Pearson’s rank correlation coefficient was 0.95. The top model successfully predicted 75% (n = 84) of the 2009 to 2012 brood locations naïve to development of the models (Fig 3). Variance inflation factors of our top models were < 5.

## Discussion

Due to the fine spatial extent (1 m) of our input image, we were able to examine influences of habitat edge on habitat selection by female sage-grouse with broods in a way that has not been done before across such a large area. Our top model included two edge-associated variables that sage-grouse appeared to avoid when selecting brood-rearing habitat: shrub/tree edge, and riparian/tree edge. As we could not differentiate between shrub types, the shrub/tree edge may have been avoided because the shrubs nearest the trees may not have been sagebrush but rather a mountain shrub community. These edge-associated variables provide more information on the relationship of selection for areas with high percent shrub and riparian and low percent tree landcover. Sage-grouse not only avoided areas with a high percentage of trees but also areas that consisted of a patchy mosaic of trees and sage-grouse habitat types at the 795 m spatial extent. In addition, sage-grouse broods were found farther from trees, representing an inverse relationship to distance to trees (Fig 2).

The variables in our top model were similar to Atamain et al. 2010 [30]. They identified “xeric mixed sagebrush” as a vegetation type that was selected during early brood-rearing and “moist sites with riparian shrubs” and “montane sagebrush” as areas that were selected during late brood-rearing. While we did not make the distinction between early and late brood-rearing habitat due to the mesic nature of our study site, we did identify percent shrub at the 795 m spatial extent and percent riparian at the 45 m spatial extent as factors selected by brood-rearing sage-grouse. We also identified a negative relationship with percent tree at the 795 m spatial extent where Atamain et al. 2010 [30] reported avoidance of pinyon/juniper woodlands. Dzialak et al. 2011 [31] showed a positive relationship between brood-rearing habitat and percent shrub at the 90 m spatial extent. Our results indicated that sage-grouse selected areas with a higher percentage of shrubs, albeit at a much larger spatial extent. Dzialak et al. 2011 [31] and Dinkins et al. 2014 [32] both showed mixed effects of distance to mesic habitat with sage-grouse showing an aversion to mesic areas during early brood-rearing and a selection for mesic areas during mid and late brood-rearing periods. Our top model did not contain distance to mesic areas. Nonetheless, we did show selection for areas with higher proportions of riparian habitat at the 45-m spatial extent.

Anthropogenic structures such as well pads have been identified as influencing habitat selection by brooding sage-grouse, but negatively influencing survival rates [18]. Well pads did not occur in our study area; however, numerous permanent structures (largely cabins) were located in otherwise suitable brood-rearing habitat. Sage-grouse with broods avoided areas close to permanent structures. The difference between our findings and previous research [18] could be due to higher human activity at the permanent structures in our area compared to well pads or some other difference between these structures and how sage-grouse perceived them.

Distance to transmission lines was another anthropogenic structure included in our top model. Sage-grouse with broods in our study area were found closer to transmission lines than random locations. One possible explanation for this is that the right-of-way, cleared for the transmission lines in our study area, may have created desirable microsite conditions for brood rearing sage-grouse. Another possible explanation is that transmission lines in our study area happened to be located in quality brood-rearing habitat and brood rearing sage-grouse did not actively avoid them. Nonetheless, transmission lines are thought to have a negative influence on sage-grouse for a variety of reasons including provision of raptor perches which has the potential to negatively influence survival rates [16]. We did not measure survival in our analyses and it is possible that there could be decreased fitness of broods that selected areas near transmission lines. A similar phenomenon has been demonstrated with other anthropogenic disturbance features. In two separate studies, sage-grouse selected areas closer to anthropogenic disturbance, but exhibited decreased fitness in these areas [18, 33].

Sage-grouse with broods selected habitat characteristics at a variety of spatial extents with at least one variable included from each of the three spatial extents we examined. The majority of these variables in the top model were best at the largest spatial extent, which reemphasizes the need to examine sage-grouse habitat selection at larger extents [16, 35–36]. However, inclusion of percent riparian at the 45-m spatial extent in the top model illustrates the importance of examining small spatial extent habitat characteristics as well. While the spectral resolution of NAIP imagery limits the specificity of the classes to broad categories (i.e. all shrubs vs. sagebrush only), the accuracy for these broad classes was sufficient to create a model that successfully predicted 75% of the 2009 to 2012 brood locations. With the success of classified NAIP imagery in this study, we suggest it may be applicable to other sage-grouse populations, species of conservation concern or any other species in habitat conducive to these methods. The accuracy of our NAIP classification was of critical importance to the validity of our model selection results. While overall accuracy of 78.5% and a kappa of 0.707 could be improved, it is within the range of other widely used vegetation classifications in the western United States (e.g., LANDFIRE = 69.3% to 87.1% overall accuracy across all western United States super zones, no Kappa reported; SWReGAP = 0.60 Kappa; SAGEMAP = 0.537 to 0.878 Kappa across all mapping zones).

The benefits or tradeoffs of using our methods with NAIP imagery are dependent upon the question of interest. The rapid and complex changes to modeling techniques as well as the availability, cost, and quality of imagery influence project applicability. Early efforts by Homer et al. in 1993 [56] demonstrated the immediate utility of remotely sensed data in mapping current-day sagebrush habitats as well as future applications in multispatial-extent modeling. Since then many others have assessed and classified habitat on a national [57], state-wide [58], or project-level [59] using medium or lower resolution Landsat imagery. One advantage to our approach was that we were able to determine the importance of edge at the patch scale on brooding female habitat selection. This may not be possible with more course resolution. Newer, more sophisticated methods combine the use of imagery, often at different spatial extents, with ancillary geospatially explicit data sets [43, 60–61], and at times, ground based verification to assess habitat change. A pitfall to our approach (using higher resolution imagery– 1m), is that currently, it cannot be used at larger scales [57–58, 60–61] due to processing time and extensive field verification. Other studies [62–63] used a similar approach to assess rangeland tree cover characteristics with relatively accurate classification and similar results. Though its efficacy may be limited to project level analyses, the classification of NAIP imagery as a base layer for landscape spatial extent analyses was a cost-effective method for examining habitat selection at varying spatial extents.

## Supporting Information

S1 File This table contains the data used to inform the habitat modeling and resource selection function processes. (CSV)
