# Correction: Assessing Greater Sage-Grouse Selection of Brood-Rearing Habitat Using Remotely-Sensed Imagery: Can Readily Available High-Resolution Imagery Be Used to Identify Brood-Rearing Habitat Across a Broad Landscape?

**DOI:** 10.1371/journal.pone.0160725

**Published:** 2016-08-01

**Authors:** Matthew Westover, Jared Baxter, Rick Baxter, Casey Day, Ryan Jensen, Steve Petersen, Randy Larsen

The key in Fig 3 was incorrectly labeled. The authors have provided a corrected figure here.

**Fig 3:**
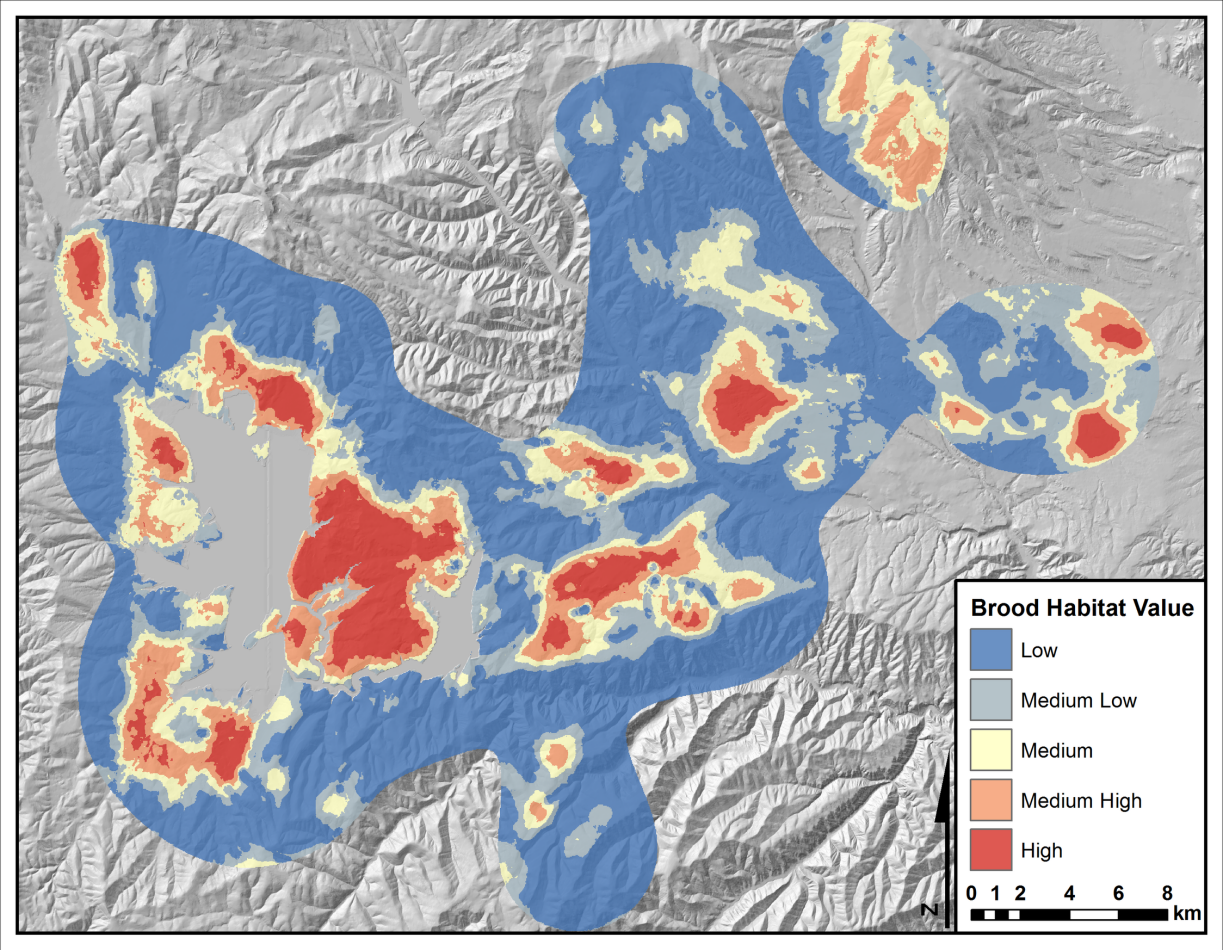
Predicted brood-rearing habitat for greater sage-grouse in Strawberry Valley, Utah based on logistic regression models of landscape features identified using 1-m NAIP imagery. Data used to generate the predictive model were collected between 1998 and 2008. Locations used to verify accuracy of the habitat map were collected between 2009 and 2012. Grey areas represent water.
